# How Social Participation Enhances “Generativity” Among Older Adults in Japan

**DOI:** 10.1093/geroni/igaf122.3011

**Published:** 2025-12-31

**Authors:** Chiharu Miura, Keiko Katagiri

**Affiliations:** Advanced Research Center for Well-being of Kobe University, Ashiya, Hyogo, Japan; Kobe University, Kobe, Hyogo, Japan

## Abstract

Japan is the world’s leading super-aged society. In contemporary Japan, older adults are no longer merely recipients of social support but have emerged as active contributors to society. They are expected to pass on their knowledge and experiences to the next generation, making generativity increasingly essential. One key factor influencing generativity in later life is social participation. Social participation is an accessible and familiar activity for older adults. It provides opportunities to foster new relationships and facilitates learning through various activities. This study examines whether new encounters, new friendships, and learning through acctivities enhance older adults’ generativity. A mail survey was conducted targeting residents aged 60-79 in an urban Japanese city (n = 1045). Out of 456 respondents, data from 299 participants engaged in social participation activities were analyzed. Serial mediation analysis using Hayes’ PROCESS macro (Model 6) was applied. The analysis revealed that ‘new friends’ and ‘learning’ fully mediated the relationship between ‘new encounters’ and ‘generativity.’ Specifically, new encounters positively influenced the acquisition of new friends, which subsequently facilitated learning, ultimately enhancing generativity. These findings suggest that new encounters play a crucial role in fostering generativity among older adults. Social participation, which generates these new encounters, is essential for the well-being and active contribution of contemporary and future older adults in society.

